# Meeting Announcement: 2nd Symposium ‘Epigenetic Inheritance: Impact for Biology and Society’ 26–28 August 2019, ETH Zurich, Switzerland

**DOI:** 10.1093/eep/dvz001

**Published:** 2019-02-07

**Authors:** Isabelle M Mansuy

**Affiliations:** Neuroepigenetics, Brain Research Institute, University and ETH Zürich

https://www.epigenetic-inheritance-zurich.ethz.ch/

The symposium will be dedicated to the theme of epigenetic inheritance, a novel discipline at the interface between biology, medicine and environmental science that studies how life experiences and environmental factors can modify the organism across generations. The symposium is a follow-up of the Latsis Symposium 2017 held at ETHZ, which raised major interest nationally and internationally and was highly successful. The 2019 symposium will gather leaders from different disciplines working on epigenetic inheritance, and cover scientific aspects from behavior to metabolism in humans and various animal models. It will feature keynote lectures from leaders in the field, and short talks from young researchers, and provide a platform for discussion and debate about the current state of research, new findings and discoveries, the challenges of the discipline and the perspectives for biology, medical research and the society.

**Main Themes:**

- Epidemiological evidence and animal models
- Reprogramming
- Transmission mechanisms
- Conceptual challenges and computational aspects of epigenetic mapping
- Impact on society and evolution

**Confirmed Speakers:**

- Patrick Allard; University of California, Los Angeles, US
- Romain Barres; University of Copenhagen, DK
- Qi Chen; University of Nevada, Reno, US
- Victor Corces; Emory University, Atlanta, US
- Lucia Daxinger; Leiden University Medical Center, NL
- Jill Escher; Escher Fund for Autism, US
- Larry Feig; Tufts University, Boston, US
- Anne Ferguson-Smith; University of Cambridge, GB
- Randy Jirtle; North Carolina State University, Raleigh, US
- Alexander Meissner; Max-Plank Institute for Molecular Genetics, Berlin, DE
- Ruth Müller; Munich Center for Technology in Society, DE
- Antoine Peters; Friedrich Miescher Institute for Biomedical Research, Basel, CH
- Andrew Pospisilik, Max-Planck Institute of Immunobiology and Epigenetics, Freiburg, DE
- Mark Robinson; University of Zurich, CH
- Julia Schröder; Imperial College London, GB
- Upasna Sharma; University of California, Santa Cruz, US
- Michael Skinner; Washington State University, Pullman, US
- Corrado Spadafora; Italian National Research Council, Rome, IT
- Azim Surani; University of Cambridge, GB

There will also be poster sessions during the meeting and a workshop ‘Meet the experts: Questions and Answers’ on the last day for students and postdocs (separate registration). You can register here: https://www.epigenetic-inheritance-zurich.ethz.ch/registration/, registration is open until 31 July 2019. Please feel free to share the information in your community and invite your co-workers, students and colleagues to participate.

Look forward to seeing you at the symposium next August.

